# An On-Line System for High Temperature Dielectric Property Measurement of Microwave-Assisted Sintering Materials

**DOI:** 10.3390/ma12040665

**Published:** 2019-02-22

**Authors:** Li Wu, Yi Zhang, Fengxia Wang, Weiquan Ma, Tian Xie, Kama Huang

**Affiliations:** 1 College of Electronic and Information Engineering, Sichuan University, Chengdu 610065, China; wuli1307@scu.edu.cn (L.W.); yizhang_ee@163.com (Y.Z.); maweiquan17@163.com (W.M.); 2 State Key Laboratory of Efficient Utilization for Low Grade Phosphate Rock and Its Associated Resources, Wengfu Group, Guiyang 550014, China; wangfengxia9999@163.com (F.W.); xietian@wengfu.com (T.X.)

**Keywords:** microwave-assisted material sintering, dielectric property, high temperature, on-line measurement

## Abstract

Microwave-assisted sintering materials have been proven to deliver improvements in the mechanical and physicochemical properties of the materials, compared with conventional sintering methods. Accurate values of dielectric properties of materials under high temperatures are essential for microwave-assisted sintering. In view of this, this paper, proposes an on-line system to measure the high temperature dielectric properties of materials under microwave processing at a frequency of 2450 MHz. A custom-designed ridge waveguide is utilized, where samples are heated and measured simultaneously. An artificial neural network (ANN) trained with the corresponding simulation data is integrated into this system to reverse the permittivity of the measured materials. This whole system is tested at room temperature with different materials. Accuracies of measuring dielectric property with an error lower than 9% with respect to theoretical data have been achieved even for high loss media. The functionality of the dielectric measurement system has also been demonstrated by heating and measuring Macor and Duran ceramic glass samples up to 800 °C. All the preliminary experiments prove the feasibility of this system. It provides another method for dielectric property measurement and improves the understanding of the mechanism between microwave and media under high temperatures, which is helpful for optimizing the microwave-assisted sintering of materials.

## 1. Introduction

Considerable interest has been drawn to the processes of materials sintering by microwave power and many potential merits have been recognized in applications [1,2,3,4,5,6]. Yang Li et al. employed microwave power to sinter the metallic matrix diamond tool bits. The relative density, the flexural strength and the abrasive ratio of the sintered sample were increased by 6.75%, 20.3% and 22.2%, respectively [7]. Application of microwave power on sintering the Cu based metallic binder conducted by Guo Shenghui et al. showed that the relative density, hardness and flexural strength increased respectively 1.25%, 3.86% and 6.28% [8]. Lai Hsuan-Lin et al. utilized microwave energy to sinter LiBa_1−x_PO4:xTm^3+^ powders. They obtained LiBa_1−x_PO4:xTm^3+^ phosphors with more uniform grain size distributions and enhanced emission intensity [9]. Leonelli Cristina et al. sintered green metal parts with microwave powers and obtained results with comparable microstructure and shape retention to the conventional sintering methods in much shorter time [10].

Unfortunately, microwave processing materials usually encounters well-known issues like hot spot and thermal runaway [11,12]. Researches have reported that accurate values of dielectric properties of materials under treating temperatures were essential for avoiding these barriers in microwave environment [13,14,15,16,17]. Moreover, the design of microwave equipment in particular demands knowledge of dielectric properties of materials under high temperatures, since they influence the microwave energy absorption ability of materials and can help avoid thermal runaway. Therefore, a thorough understanding of dielectric properties at elevated temperatures is necessary.

Researchers have investigated the designs of high-temperature permittivity measurement systems. M. Arai et al. have developed two techniques to obtain permittivity measurements up to about 1200 °C [1]; Z. Li employed a cylindrical resonant cavity to measure dielectric property of metallurgy materials [17]; G. F. Guo et al. proposed a test system based on short-circuited line method to measure the complex permittivity of low-loss materials [18], to name a few. However, a lot of the existed measure methods raised the temperatures of materials with a furnace or electric heating elements [17,18,19,20,21,22]. Once the desired temperature was reached, the holder with materials inside was moved to the cavity for dielectric measurement and then rapidly moved back to the furnace. Some cooling of the sample occurred during transfer from the furnace to the cavity, which posing negative impacts on the accuracy of the measurement. Besides, these methods were tedious and not likely to be used when a large number of samples were to be measured under dynamic temperature conditions in the high temperature area, where conventional heating techniques were hard to control.

In view of these disadvantages, some researchers proposed to heat the measurement device filled with samples, raising the temperature of tested material by heat conduction [18,20,23,24]. This means enabled the continuous measurement. However, the equipment presented a much higher requirement on the quality of metal material, which increased the cost of this measured system. Moreover, the metal device and the tested sample would experience thermal expansion in the heating process, leading to a shift of their relative locations. Since the thermal expansions were hard to express precisely, errors would be difficult to be corrected.

D. Couderc [25] and José M. Catalá-Civera [26] respectively developed a dual-mode cylindrical cavity to realize on-line high-temperature permittivity measurement, with one mode for heating samples directly and another for measurement. The same principle was also adopted in an innovative design with two parallel waveguide arms [27]. This technique not only solved the problem of high cost and achieved real-time measurement but also reduced the thermal expansion of measure device and the errors it caused.

This paper, to reduce the complexity of dual operation, proposes the transmission/reflection method to determine the dielectric properties of materials under high temperatures in real time, due to the system using transmission/ reflection method is simpler, easy-controlling and more accurate [28]. A custom-designed ridge waveguide with single mode (TE_10_ at 2450 MHz) was utilized, where heating and measuring were performed simultaneously. An artificial neural network trained with the corresponding simulation data was used to reverse the permittivity. The whole system was tested at room temperature with different materials. Measurements on dielectric properties of ceramic and glass were also performed in order to evaluate the performance of the setup under high temperatures. The preliminary experiments prove the feasibility of this system.

## 2. System Design

**Figure 1:**
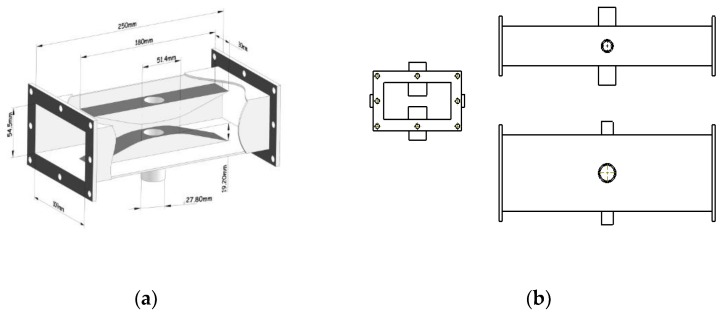
Schematic of ridge waveguide: (**a**) 3-D structure; (**b**) Three views.

Ridge waveguide can focus the electromagnetic energy in a small space between two ridges, which makes it capable of heating the sample quicker and more sensitive to the permittivity change of the material under test (MUT). Moreover, since the space between two ridges for placing MUT is narrower, this design needs less sample amount in each measurement. Therefore, a ridge waveguide (based on the BJ22 waveguide) is designed and chosen here as the heating and measuring part. Its schematic photo is shown in Figure 1. Two identical ridges are designed respectively on the centers of top and bottom surfaces of the waveguide to focus the electric field between them. One can also observe that the ridges consist of three parts: a platform and two metal slopes. The slopes act as stepped impedance transformers to make the impedances of regular waveguide and ridge waveguide matched. Two holes are dug through the platforms for placing the sample-partially-filled quartz tube. The dimensions and positions of the platforms, holes and quartz tube are optimized to make this equipment more sensitive to different tested materials and capable of heating samples quicker and more uniformly. The ridges are designed specially shorter than the waveguide for the higher modes stimulated by the discontinuousness of the structure to be dissipated and avoiding their adverse impacts on the measurement.

Other two cut-off waveguides are introduced on the side surfaces of ridge waveguide for the observation of sample change during the heating. The dimensions and positions of access holes in the waveguide are designed to ensure that they do not disturb the field inside and to prevent microwave leakage. The thickness of metal walls is 2 mm.

**Figure 2:**
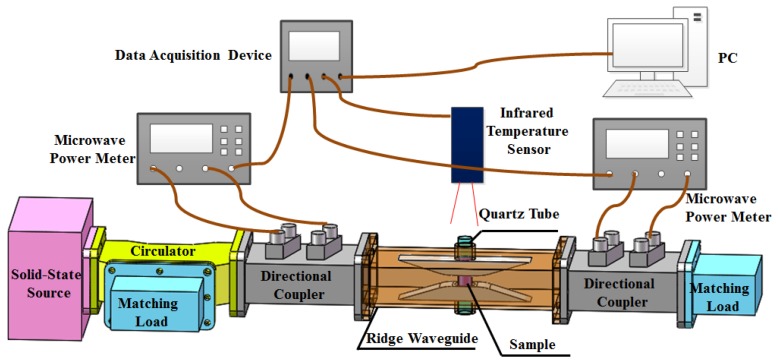
Schematic of the experiment setup.

Figure 2 shows the schematic of the experimental system. Two double directional couplers are connected to two ends the of ridge waveguide to measure the input power, reflection power and transmission power with calibrated microwave power meters. A microwave solid state source is employed to provide heating energy. To protect the source, a circulator with a water load is employed to link the microwave source and one double directional coupler. A matched load locates at the other end of this system. Sample is filled in a quartz tube placed in the ridge waveguide. An infrared thermometer is applied to measure the surface temperature of tested material through the top cut-off waveguide, while one side hole is used to place a video camera to observe the dielectric sample during processing. Water cooling is added around the ridge waveguide to cool it down during the high temperature measurement. To make this system more user friendly, a data collecting module (a data acquisition device and a PC) is used to record the measured data and reverse the dielectric properties of materials automatically.

## 3. Methodology

Success of permittivity measurement depends upon the theoretical model accurately relating the measured quantities to the complex permittivity of the sample. However, since the custom-designed ridge waveguide is not a standard element, the theoretical relation between measured S parameters and complex permittivities for waveguide is not reliable any more here. Modern approaches extend into modeling and an optimization technique based on artificial neural networks (ANNs) [29,30]. This technique does not apply any limitations on the shape of the measured equipment or on the field distribution [31,32,33]. Besides, ANNs can work with numerical measurement/simulation data, are suitable for dealing with optimization-type problems and can handle nonlinearities (which we expect to find in simulated and measured S parameters) much better than polynomial approximations [32]. Therefore, FDTD (finite-difference time-domain) modeling and an ANN were applied in this paper to reverse the complex permittivities of tested materials.

The models of this ridge waveguide with different dielectric inclusions in a cylindrical quartz tube were simulated at 2450 MHz to obtain the corresponding S parameters.

**Figure 3:**
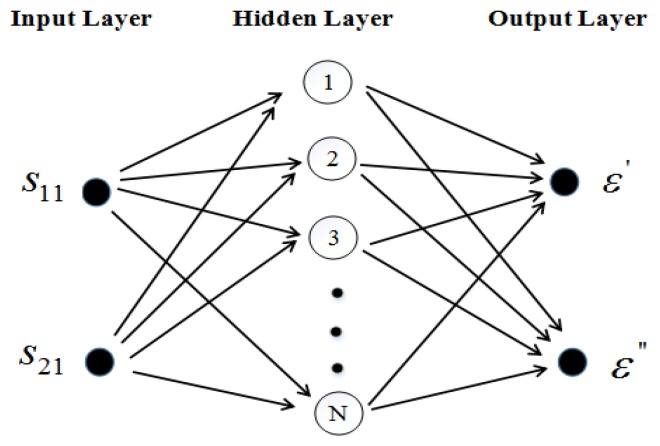
Schematic architecture of neural network.

It may encounter the problem of multi-value when using ANN to do the inversion: different complex permittivity corresponds the same or very close S parameters. This will lead to wrong measured results. To solve this problem, the ridge waveguide model was optimized to get one-to-one correspondence from complex permittivity to S parameters. With the simulated data, a two-input-two-output ANN was constructed to create the relation network between $S_{11}$, $S_{21}$ and $\varepsilon^{\prime }$, $\varepsilon^{\prime \prime }$. The network structure is shown in Figure 3.

The input of the network is an N × 2 matrix

(1) $$X=\left[\begin{array}{c} \begin{array}{c} {\left(S_{11}\right)}_{1}\;{\left(S_{21}\right)}_{1,}{\left(S_{11}\right)}_{21}\;{\left(S_{21}\right)}_{2,}\ldots ,\\ {\left(S_{11}\right)}_{n}\;{\left(S_{21}\right)}_{n} \end{array} \end{array}\right]$$

It contains all the values of simulated reflection coefficients and transmission coefficients at frequency of 2450 MHz. The output of network is thus the N × 2 matrix of corresponding $\varepsilon^{\prime }$ and $\varepsilon^{\prime \prime }$. The complex permittivity could be obtained with the following formulas [34,35]:

(2) $$\varepsilon^{\prime }=f_{2}\left(\overset{N}{\underset{j=1}{\sum }}w_{j1}f_{1}\left(w_{1j}\left|S_{11}\right|+w_{2j}\left|S_{21}\right|-\theta_{j}\right)-\theta_{1}^{\prime }\right)$$

(3) $$\varepsilon^{\prime \prime }=f_{2}\left(\overset{N}{\underset{j=1}{\sum }}w_{j2}f_{1}\left(w_{1j}\left|S_{11}\right|+w_{2j}\left|S_{21}\right|-\theta_{j}\right)-\theta_{2}^{\prime }\right)$$

In the formula, $w_{j1}$ and $w_{j2}$ are respectively the weights of $\left|S_{11}\right|$ and $\left|S_{21}\right|$ connecting to the jth neurons. $w_{1j}$ and $w_{2j}$ show the weights from jth hidden neurons to $\varepsilon^{\prime }$ and $\varepsilon^{\prime \prime }$, respectively. N denotes the number of hidden neurons in the network. $\theta_{j}$ is the threshold value of the jth neuron in hidden layer. $\theta_{1}^{\prime }$ is the threshold value of $\varepsilon^{\prime }$, while $\theta_{2}^{\prime }$ is the threshold value of $\varepsilon^{\prime \prime }$. $f_{1}$ represents the transfer function of the hidden layer, while $f_{2}$ denotes that of the output layer.

Normally, for a given input cell $X_{k}$, there will be errors between the desired and actual outputs of the ANN, which can be expressed as

(4) $$e_{\varepsilon^{\prime }}=\frac{1}{2}\overset{P}{\underset{k=1}{\sum }}{\left|\varepsilon^{\prime }\left(X_{k},\;w\right)-E_{k}^{\prime }\right|}^{2}$$

(5) $$e_{\varepsilon^{\prime \prime }}=\frac{1}{2}\overset{P}{\underset{k=1}{\sum }}{\left|\varepsilon^{\prime \prime }\left(X_{k},\;w\right)-E_{k}^{\prime \prime }\right|}^{2}$$

where $\varepsilon^{\prime }\left(X_{k},\;w\right)$ and $\varepsilon^{\prime \prime }\left(X_{k},\;w\right)$ are the output of ANN for input $X_{k}$, while $E_{k}^{\prime }$ and $E_{k}^{\prime \prime }$ represent the desired outputs. P is the number of training cells.

**Figure 4:**
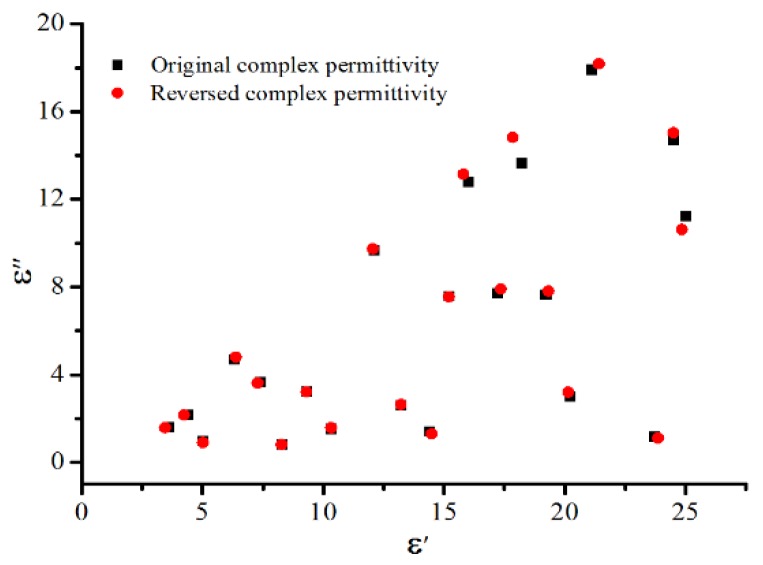
Comparison of the original and reversed complex permittivity.

The errors depend on the training method as well as on the number of hidden neurons. To optimize the network and minimize the errors, the number of neurons was determined by using a simple trial-and-error process and the backpropagation technique. It appeared that the network with 10 or more hidden neurons generated very acceptable results. With the numerical testing, the parameters for training the network were chosen like those shown below in Table 1. It is seen that the sum-squared error of the network is of order of 10^−3^. To check the reverse error for certain inputs, random data (S_11_, S_12_) were also picked to reverse their corresponding complex permittivity. Parts of the original and reversed complex permittivity are demonstrated in Figure 4. It is obvious that the reverse error for different data varies. The maximum error for dielectric constant is about 4.5%, while that for loss factor is around 9% when the loss tangent is very high.

This solution has been programed. A module of data collection was coded and applied to simultaneously acquire the information of temperature and S parameters. Integrating the ANN code into this data collection module, we programed a graphical user interface (GUI) interactive interface to realize auto measurement, making this system friendly to users.

## 4. Measured Results and Discussion

The accuracy of reversed dielectric property reached by the transmission /reflection method with a custom-designed ridge waveguide must be evaluated first. To verify the accuracy of the tested dielectric property, a number of different samples in wide range of dielectric constants and losses were measured at room temperature with the measurement system described above. All the samples were filled in a 2.2 mm thick quartz tube with inner diameter of 18 mm in the experiments. Results were compared with the theoretical ones or those measured by other researchers. The comparisons are tabulated in Table 2. Measurements under dynamic temperatures were also conducted to verify the feasibility of this system to do the on-line high-temperature measurement.

### 4.1. Room Temperature Measurement

The easy-to-access analytical pure reagents like methanol, ethanol and different concentrations of methanol-ethanol mixtures and methanol-n-butanol mixtures were measured separately at room temperature. All the measured data were compared with those obtained by others [36] and listed in Table 2. The reference permittivity of mixtures was calculated according to the Bruggeman formula [37].

It is obvious that the accuracy of the ridge waveguide system is quite good, with the maximum discrepancies between theoretical and measured results less than 9% even for high loss materials. It seems that the accuracies of measurements for dielectric constant larger than 14 are respectively higher, while those for the dielectric loss show a wavy tendency. One can also observe that the estimation of dielectric properties of single reagent is better than that of reagent mixtures. Researchers reported that the conventional mixture formulas cannot describe the dielectric property of mixed reagents appropriately, since the mixture may present an anomaly in its dielectric property [38,39]. There may be also an anomaly in the mixtures measured in the paper, causing the difference between the measured and calculated data of mixtures.

Many other factors may cause the measured errors. During the experiment, we found that the heights of samples were strongly related to the measured S parameters (dielectric property). The higher the loss tangent is, the stronger dependence of S parameters (dielectric property) is on the height of sample. Therefore, one of the main source introducing errors in this measurement system may lay on the small variation of height of sample in experiments. Another main error source may be the trained ANN. Normally, there exist difference between the original data and the reversed data obtained by the trained ANN, due to the non-infinite data used for training. This is already revealed in the last section. Different range of permittivity may need different density samples to train the network to guarantee the reverse accuracy. This partially expresses why the measured accuracies for different dielectric constant and loss factor ranges alter.

The accuracy of permittivity measurement at high temperature by this system is estimated to be very close to that of the measurement at room temperature, because the thermal expansion of the testing waveguide is negligible due to the thermal cooling and the rapid measurement. Besides, it is reported that the dielectric property of quartz tube remain practically unaltered under 1000 degrees [18,40], which will not pose any negative impacts on the measured results.

### 4.2. High Temperature Measurement

The final goal of this paper is to propose an on-line high-temperature dielectric property measurement system. Its feasibility of permittivity measurement under dynamic temperatures must be checked. The temperature dependence of dielectric property of Macor glass and Duran glass samples were measured in this section to test the performance of the method aforementioned. An infrared temperature sensor was used to measure the temperature changes of the samples through the top hole. It is difficult to obtain the relatively accurate bulk temperature of the sample by multi-point measurement in microwave environment. Since the sample size is very small, its temperature distribution under conventional heating is also relatively uniform, as that under microwave heating. The bulk temperature of media with microwave heating is thus calibrated with the measured results of the conventional heating. Samples having similar thermal properties were heated by conventional heating, with the top surface of the sample exposing to the ambient. Three thermocouples were employed to measure the temperatures of the top surface, side surface and the center of the sample. The temperatures of side surface and center were averaged to obtain the bulk temperature of the sample since the sample size was small and its temperature distribution was approximately uniform. A relation between the bulk temperature and the top surface temperature under conventional heating was established, which was then applied to reverse and estimate the bulk temperature of a sample with the top surface temperature under microwave heating. Rod-shape Macor glass sample and Duran glass fragment sample were respectively filled in a quartz tube and placed between two ridges. The measured consequences are compared with those obtained by M. Arai et al. [41] and José M. Catalá-Civera et al. [26]. Since the maximum temperature of Macor glass is around 1000 degrees and the Duran glass will melt when its temperature reaches 860 degrees [26], the experiments of permittivity measurement are conducted only from room temperature to 800 degrees.

**Figure 5:**
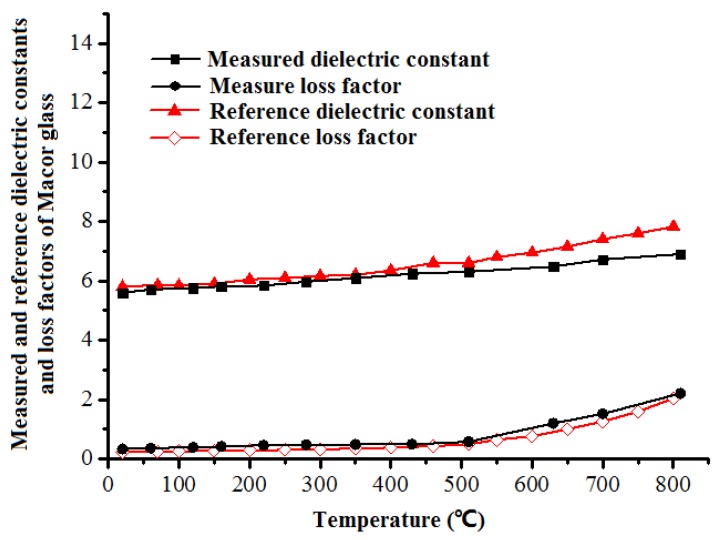
Comparison of measured and reference dielectric properties of Macor glass.

It is seen from Figure 5 that the dielectric constant of Macor increases moderately with increasing temperature and the growth rate rises when its temperature reaches 600 °C. The dielectric loss factor follows a similar pattern. The measured dielectric property of Macor is compared with those obtained by Arai et al. [41] for the same material. It reveals good agreement between these two methods, especially at low temperatures, which shows the feasibility and accuracy of this ridge waveguide based system under dynamic temperatures.

**Figure 6:**
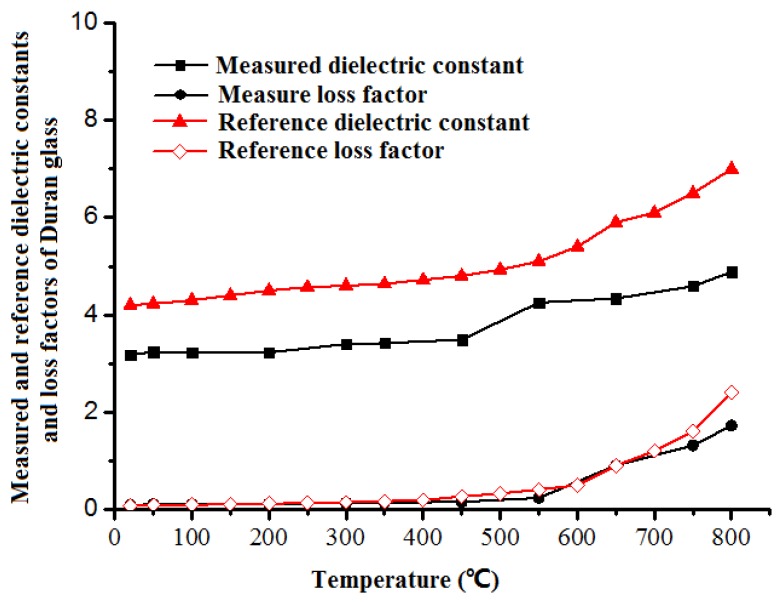
Comparison of measured and reference dielectric properties of Duran glass.

To double check the system, Duran glass fragment was employed to measure its relative permittivity, since we cannot find the solid rod Duran sample. The black lines in Figure 6 show the relative complex permittivity of the Duran Glass sample measured by the ridge waveguide. One can observe that there exist differences between our measured results and the reference data, especially in the dielectric constant. However, its rationality still can be addressed.

The main reason to cause the discrepancy is that the sample used in our paper is Duran glass fragment, while that in the reference paper is in solid rod. The sample is in fact a mixture with majority of glass fragment and a little of air. According to the Bruggeman formula, their dielectric property is between the pure Duran glass and air, which accords very well the comparison shown in Figure 6. What’s more, the change tendency of the mixture is similar to the reference one. Its dielectric constant, as represented in Figure 6, undergoes a smooth monotonic augment with increasing temperature and a markedly rise when temperature reaches 500 °C. On the other hand, the dielectric loss factor commences with a very low value but increases rapidly at temperatures beyond 550 °C. It is reported that the Duran glass will experience transition in vicinity of 525 °C, making the dielectric property slope shows anomaly around the glass transition [26]. The measured data in this paper conform to the theoretical dielectric property change, which is also a proof of the accuracy of the measured data and the availability of this waveguide system.

In both experiments, no size changes in the dielectric samples were observed during heating. Therefore, the accuracy of dielectric calculations, as stated in room temperature measurement section, is estimated to be less than 9% for the dielectric property in the entire measured range.

## 5. Conclusions

In this paper, to obtain the dielectric property variation of microwave sintering materials, an on-line high temperature dielectric property measurement system at frequency of 2450 MHz is successfully established. This system mainly consists of two parts: a ridge waveguide where samples are heating and measuring simultaneously; a trained artificial neural network (ANN) for reversing the dielectric property of measured samples. A module of data collection was integrated into this system to realize auto measurement, making this system user friendly. The functionality of the dielectric measurement system has been demonstrated by heating and measuring two different glasses ceramic samples up to 800 °C. Accuracies of the permittivity obtained by this ridge waveguide based system have been verified by comparing measured results of different materials with those acquired by other researchers.

This system provides another way to measure the dielectric property of materials at high temperature, which may help improve the understanding of the mechanism between microwave and media, design microwave devices for materials sintering and facilitate comparisons with conventionally heated materials.

## Figures and Tables

**Table 1:** Parameters used to train the artificial neural networks (ANN).

Study Ratio	Maximum Iteration Times	Target Accuracy ( $e_{\varepsilon^{\prime }},e_{\varepsilon^{\prime \prime }}$)	Hidden Neuron	Hidden Layers
0.01	1000	0.005	15	1

**Table 2:** Comparison of measured and reference dielectric properties of several samples at room temperature.

Media	Measured $\varepsilon^{\prime }$	Reference $\varepsilon^{\prime }$	Errors	Measured $\varepsilon^{\prime \prime }$	Reference $\varepsilon^{\prime \prime }$	Errors
Ethanol	9.70	8.94	8.50%	7.57	7.60	0.39%
Methanol	25.80	24.97	3.30%	13.93	14.48	3.80%
4methanol + 1ethanol	19.95	21.03	5.10%	13.57	13.04	4.06%
2methanol + 3ethanol	14.14	14.09	0.35%	11.03	10.14	8.78%
4methanol + 1N-butanol	17.95	19.17	6.40%	10.77	11.12	3.15%
2methanol + 3N-butanol	9.89	9.13	8.30%	5.84	5.39	8.35%

Note: “4methanol + 1ethanol” means the volume rate between methanol and ethanol is 4:1.
